# Characterization of Lipophorin Receptor (LpR) Mediating the Binding of High Density Lipophorin (HDLp) in the Silkworm, *Bombyx mori*

**DOI:** 10.1673/031.011.15001

**Published:** 2011-11-08

**Authors:** G. Ravikumar, K. V. Vardhana, H. K. Basavaraja

**Affiliations:** Seri-biotech Research Laboratory, Central Silk Board, Carmelaram Post, Kodathi, Bangalore 560035, India

**Keywords:** Immunoblotting, ligand binding

## Abstract

In an earlier report, we described the gene encoding a lipophorin receptor (LpR) of the silkworm, *Bombyx mori* L. (Lepidoptera: Bombycidae), and recombinant expression of the protein. The present study was performed to characterize the corresponding native *Bm*LpR and its binding characteristics. Polyclonal anti-LpR antibody prepared against the cloned receptor fragment from the cytoplasmic domain specifically detected the receptor. Through immunoblotting, ovary and brain membrane protein samples of *Bm*LpR have shown an apparent molecular mass of 105 kDa and 120 kDa under nonreducing and reducing conditions, respectively. Ligand binding of LpR supported the immunoblot results. It bound to high density lipophorin (HDLp) and has shown requirement of Ca^2^ in binding. Further, a dose-dependent inhibition by EDTA was observed in receptor ligand binding. The characteristics of the *Bm*LpR protein confirm the properties of a ligand-receptor interaction similar to that of vertebrate low density lipoprotein receptor (LDLR).

## Introduction

Receptor mediated endocytosis of plasma lipoproteins play crucial roles in the metabolism of fats and cholesterol (Goldstein et al. 1985). The low density lipoprotein receptor (LDLR) is the most extensively studied lipoprotein receptor through genetic, biochemical and molecular analysis. In vertebrates, the LDLR family comprises seven core members including the very low density lipoprotein receptor (VLDLR) and several distantly related genes (Herz 2001). Many of these proteins have multifunctional roles other than lipid clearance, and novel unexpected functions are being discovered at rapid rates (Herz and Bock 2002; Herz et al. 2009). Compared to vertebrates, insects are underrepresented in lipid metabolic studies. Lipophorin (Lp) is the major hemolymph lipoprotein in insects and functions as a reusable shuttle that accepts and delivers lipids from one tissue to another (Law and Wells 1989; Van der Horst et al. 2005). In many insects Lp acts as one of the most important yolk protein precursors. The lipophorin receptor (LpR) on the cell surface binds to Lp and is sequestered through receptor-mediated endocytosis. Larval fat body cells have been demonstrated to internalize high density lipophorin (HDLp) by means of receptor mediated endocytosis in *Manduca sexta* and *Locusta migratoria* (Tsuchida and Wells 1990; Dantuma et al. 1999). Additionally, several insect LpRs have been characterized at the molecular level (review: Tufail and Takeda 2009). However, little is known regarding the binding characteristics of LpR and its ligand, Lp.

An LpR from the silkworm, *Bombyx mori* L. (Lepidoptera: Bombycidae), was recently cloned and characterized, and found to be homologous to vertebrate very low density lipoprotein receptor (VLDLR), belonging to LDLR super family (Gopalapillai et al. 2006). The study indicated the presence of four isoforms derived from a single gene by alternative splicing and was designated as LpR1, LpR2, LpR3 and LpR4. The LpR1 seemed to be a full receptor as it had an addition of 27 amino acids in the glycosylation domain and was expressed in more tissues compared to other variant forms. This report uses LpR terminology instead of LpR1–LpR4. Although the molecular characterization of *Bm*LpR including its recombinant protein expression were studied in detail, the lack of data on endogenous protein and binding characteristics make the study incomplete. This study aims to fill the knowledge gap by identifying a native form of *Bm*LpR and functionally characterizing it through ligand binding assays.

## Materials and Methods

### Lp Collection

Hemolymph from fifth instar larval *B. mori* (day 5) was collected in PBS with pH 7.4. Hemocytes were removed by centrifugation at 20,000 × g for five minutes. Potassium bromide (KBr, 0.44 g/ml) was added to the supernatant, overlaid with 0.9% NaCl and centrifuged at 50,000 rpm (Beckman 70.1 TI, www.beckmancoulter.com) for 16 hours at 4 C. HDLp (d = 1.0635 g/ml), which formed a clear yellow band, was collected, desalted, and used immediately for the binding assay. Protein estimation was performed with the BCA protein assay kit (Pierce, www.piercenet.com) using BSA as standard.

### Preparation and Solubilization of Membrane Proteins

Ovary and brain from pupa (day 5–7) were dissected out and homogenized in ice cold extraction buffer (20 mM, Tris HCl, 150 mM NaCl, 2 mM CaCl_2_, pH 7.4) containing protease inhibitor mixture (Amresco, www.amresco.com). The homogenate was centrifuged at 1000 × g for 10 minutes, supernatant was filtered and again centrifuged at 800 × g for 10 minutes. The membrane preparations were then pelleted by centrifugation at 20,000 × g for three hours and resuspended in extraction buffer at a concentration of 10 mg protein/ml containing protease inhibitor mixture. The suspension was sonicated for 15 seconds at micro-probe setting 5 (Sonic Vibra Cell, www.sonics.com) and diluted with an equal volume of 2% Triton X-100 in the same buffer. After mixing for one hour at 4 C, insoluble material was removed by centrifugation at 20,000 × g for 10 minutes.

### Immunoblot

Extracted membrane proteins were separated on 7.5 % SDS-PAGE gels under nonreducing and reducing conditions (Laemmli 1970) and then electrophoretically transferred using a TE 77 Semi dry transfer unit (Amersham, www.gelifesciences.com) to polyvinylidene difluoride membranes (Hybond, Amersham). Blots were probed with rabbit anti-5mLpR antibody, produced from a synthetic peptide AQEPLNKPNTEFV obtained from the cytoplasmic tail of LpR1–3. Bound antibodies were detected with alkaline phosphatase conjugated goat anti-rabbit IgG and 5-bromo-4-chloro-3-indolyl-phosphate/nitro blue tetrazolium (BCIP/NBT, Western MAX, Amresco). Incubation conditions of antibodies, washing procedures and subsequent steps were according to manufacturer's instructions.

### Ligand Binding

The membrane proteins were prepared from the ovary and blotted as above. After blocking, the membrane was incubated with 20 µg/ml HDLp in binding buffer (20 mM HEPES, 150 mM NaCl, and 2 mM CaCl_2_, and 0.5% BSA at pH 7.5). After extensive washing with the above binding buffer (minus BSA), the blots were incubated with rabbit anti-HDLp antibody prepared against apolipophorin I & II of *Bm*HDLp. Bound antibodies were detected with alkaline phosphatase-conjugated goat anti-rabbit IgG and BCIP/NBT as above. Incubation conditions of antibodies, washing procedures and subsequent steps were followed according to manufacturer's instructions.

**Figure 1.**
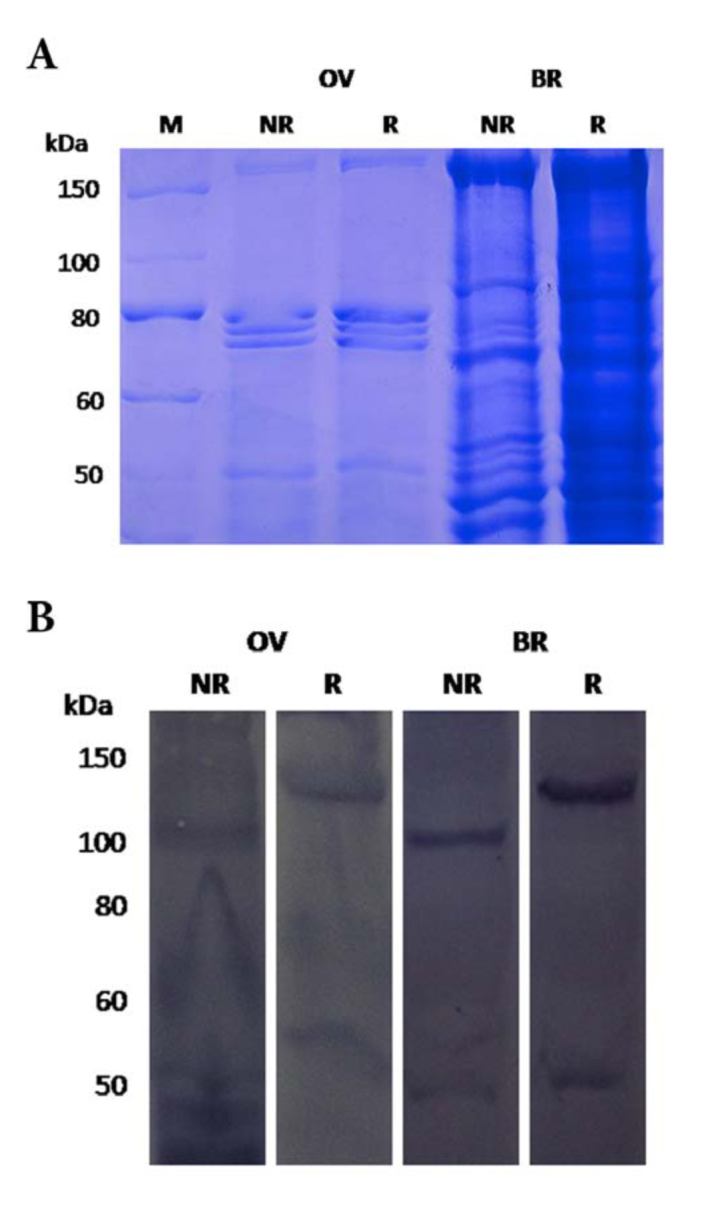
((A) Profile of whole membrane protein preparations (40– 60 µg) analysed on a 7.5 % gel. OV, ovary; BR, brain; M, molecular weight markers; NR, nonreducing conditions; R, reducing conditions. (B) lmmunoblot analysis of endogenous *Bm*LpR. Solubilised membrane preparations from ovary (OV) and brain (BR) were subjected to immunoblot analyses using anti-LpR antibody. Receptor bands of 105 kDa and 120 kDa were detected under nonreducing (NR) and reducing (R) conditions respectively. M, molecular weight markers. High quality figures are available online.

## Results

A SDS-PAGE profile of the membrane proteins is shown in Figure 1A. The proteins were transferred to a polyvinylidene fluoride (PVDF) membrane and followed by immuoblotting. Immunoblot with an anti-*Bm*LpR antibody detected a protein of apparent molecular mass of 105 kDa and 120 kDa under non-reducing and reducing conditions, respectively (Figure 1B). Membrane preparations from ovary and brain samples showed the same results. Since the amount of receptor protein which can be obtained in the solubilised membrane preparations was very small, the specific band of the receptor was not visualized in the Coomassie Brilliant Blue R250-stained gels, though it was clearly detected in the Western blot. The presence of a low molecular weight protein band in the blots may have resulted from non-specific binding of antibodies or degradation of the receptor protein.

**Figure 2.**
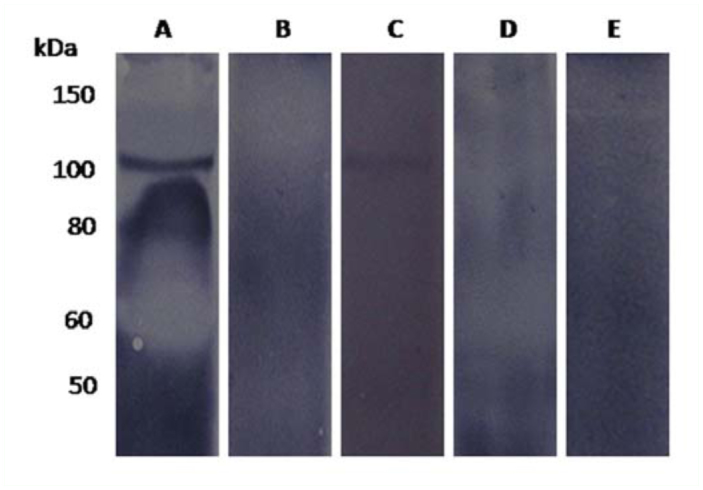
Ligand blotting analysis to reveal *Bm*LpR protein binding to HDLp. (A) Receptor binding in the presence of Ca^2+^; (B) without Ca^2+^ (in the absence of Ca^2+^ in extraction and binding buffer); (C) receptor binding in the presence of 0.5 mM EDTA; (D) with 5 mM EDTA; (E) receptor binding under reducing conditions. M, molecular weight markers. High quality figures are available online.

In order to identify whether the 105 kDa membrane protein was specific to HDLp, ligand binding was carried out. It showed 105 kDa receptor protein bound to HDLp, and a single band was detected using *anti-Bm*HDLp antibody (Figure 2). However, the low molecular weight protein band(s) which appeared in the immunoblot was not detected in the ligand blot. It clearly establishes the fact that the ovary contains high affinity and specific binding sites for HDLp and the ligand blot has required specificity to detect the receptor. On the other hand, the binding was not detected under reducing conditions, suggesting the requirement of intact cysteine residues for ligand binding (Figure 2E). The ligand binding domain of *Bm*LpR, like most insect and vertebrate lipoprotein receptors, has eight cysteine-rich repeats (Gopalapillai et al. 2006). Calcium is required for receptor ligand interactions (Dirlam et al. 1996) and precise role of this is not fully understood. The LpR receptor contains two types of cysteine-rich repeats that would be predicted to bind calcium, the ligand binding repeats and the epidermal growth factor (EGF)-like repeats. To elucidate this, calcium divalent cation (Ca^2+^) and its chelating agent EDTA were used in the study. The presence of Ca^2+^ in the binding was necessary, whereas no binding was observed in the absence of Ca^2+^ (Figure 2A, 2B). No quantification of Ca^2+^ required for the binding was carried out. However, 2 mM Ca^2+^ present in the binding buffer was sufficient for the binding. In addition, the binding was inhibited by 5 mM EDTA, whereas 0.5 mM of EDTA did not inhibit binding (Figure 2C, 2D).

## Discussion

The *Bm*LpR using immuno and ligand blots were analyzed. A protein of apparent molecular mass of 105 kDa was detected which bound to anti-*Bm*LpR antibody. The specificity of the receptor visualized by the immunoblot was indicated by the demonstration of HDLp binding protein of the same molecular weight in ligand blotting assay. Biochemical properties of the *Bm*LpR are very similar to LDL/VLDL receptors and other insect LpRs (Schneider et al. 1982; George et al. 1987; Tufail and Takeda 2009) in several aspects. All have apparent molecular mass between 95kDa–140 kDa as determined by SDS-PAGE under nonreducing conditions followed by ligand binding assay: human receptor = 130 kDa (Battey et al. 1994); chicken oocyte receptor = 95 kDa (George et al. 1987); *Manduca sexta* receptor = 120 kDa (Tsuchida and Wells 1990); *Aedes aegypti* receptor = 140 kDa (Cheon et al. 2001); *Locusta migratoria* receptor = 110 kDa (Van Hoof et al. 2003); *Galleria mellonella* receptor = 97 kDa (Lee et al. 2003a, 2003b). However, unlike vertebrate lipoprotein receptors which show considerable cross species ligand binding ability (George et al. 1987), insect LpR did not bind to human low density lipoprotein (Tsuchida and Wells 1990). This may be explained by significant receptor-ligand affinity in the diacylglycerol content of insect Lp, as well as observed structural differences the lipid moiety between mammalian and insect lipoproteins (Van der Horst and Ryan 2005).

The role of cysteines and disulphide bonds in ligand recognition is evident under reducing conditions as the binding of the LpR to Lp did not take place in the presence of reduction. Reduction of disulphide bonds by reducing agents destroys the structure and abolishes the binding (Goldstein and Brown 1974). The molecular mass difference in reducing and non-reducing conditions is due to the cysteine-rich domains that migrate considerably faster under non-reducing conditions as compared to reduced state, when their disulphide bonds become unfolded, exposed, and bulkier. The predicted molecular mass from the deduced amino acid sequence of *Bm*LpR is approximately 100 kDa (Gopalapillai et al. 2006) and the apparent molecular mass of the endogenous receptor under reduced state is 120 kDa. This size difference may be linked to the posttranslational modifications of the receptor especially glycosylation. To identify the receptor we have used an antibody available against the cytoplasmic tail sequence of LpR 1–3; the molecular mass of all *Bm*LpR isoforms are too close to each other to be differentiated in the blots. However, out of four isoforms, the LpR1 was present in most tissues with high transcript levels in ovary and brain, and dominant over other isoforms (Gopalapillai et al. 2006). This may indicate that the receptor identified in the present study is LpR1.

It is well known that the ligand binding domain (LBD) of LDLR binds to Ca^2+^ which is critical for correct folding and disulphide formation of LBD and for the binding of ligands to LDLR (Goldstein and Brown 1974; Daniel et al. 1983). Our results are consistent with the requirement for Ca^2+^ as the binding of *Bm*LpR to HDLp was inhibited by the absence of Ca^2+^. Although Ca^2+^ was required for the binding, increasing amounts of Ca^2+^ have been shown to be inhibitory in *G. mellonella* (Lee et al. 2003a). However, in *M. sexta* the requirement of Ca^2+^ in binding was shown to be essential in one study (Tsuchida and Wells 1990), but was shown as not required in another (Gondim and Wells 2000). In *Bm*LpR, a concentration dependent inhibition of receptor binding was observed using EDTA as 0.5 mM of EDTA did not inhibit the binding, whereas 5 mM of EDTA had an inhibitory effect. These results are consistent with *M. sexta* and *G. mellonella* LpRs, and in the former case the inhibition by EDTA was reversed by the addition of Ca^2+^ in the incubation medium (Tsuchida and Wells 1990; Lee et al. 2003a). Ca^2+^ induces a conformational change in the LBD of the LDL receptor and maintains the cysteine-rich regions in a more folded and native state (Kimberly et al. 1998). Taken together, these data suggest that Ca^2+^ gives stability in structure of LpR, while EDTA is disruptive, resulting in loss of ligand binding. Suramin, a polysulfated polycyclic hydrocarbon has been shown to inhibit binding of various ligands such as vertebrate LDL and insect HDLp (Schneider 1982; Tsuchida and Wells 1990).

The properties of the *Bm*LpR described here are similar to LDLR/VLDLR and fulfill the characteristics expected of a lipoprotein receptor. The quantitative and competitive binding data of the receptor will further strengthen the present findings. Recent reports from mammalian lipoprotein receptors show diverse functions not only in systemic clearance of lipoproteins, but also in signal transduction, brain development, and other important physiological functions (Herz and Bock 2002; Herz et al. 2009). Future research could investigate whether insect LpR binds to ligands other than Lp; these possibilities are currently being explored.

## Abbreviations:

LpR: lipophorin receptor;
Lp: lipophorin;
HDLp: high density lipophorin;
LDLR: low density lipoprotein receptor
VLDLR: very low density lipoprotein receptor;
LBD: ligand binding domain
